# Temporal and spatial analysis of notifications of sexual violence against male children and adolescents in Brazil, 2013 to 2022: an ecological study

**DOI:** 10.1590/S2237-96222024v33e20231439.en

**Published:** 2024-10-14

**Authors:** Beatriz Caroline Leão Lima, Cássio Eduardo Soares Miranda, Fernando Ferraz do Nascimento, Jesusmar Ximenes Andrade, Malvina Thais Pacheco Rodrigues, José Wicto Pereira Borges

**Affiliations:** 1 Universidade Federal do Piauí, Programa de Pós-Graduação em Saúde e Comunidade, Teresina, PI, Brazil

**Keywords:** Violencia Sexual, Abuso Sexual de Niños y Adolescentes, Estudios Ecológicos, Estudios de Series Temporales, Análisis Espacial, Sex Offenses, Child Abuse, Sexual, Ecological Studies, Times Series Studies, Spatial Analysis

## Abstract

**Objective:**

To analyze the temporal trend and the spatial distribution of reported cases of sexual violence against male children and adolescents, and their relationship with municipal development in Brazil.

**Methods:**

This is an ecological study with data from the Notifiable Health Conditions Information System and the Municipal Human Development Indexes (MHDIs), from 2013-2022. Prais-Winsten regression was used for temporal analysis and the Moran Index for spatial analysis.

**Results:**

There were 39,967 reports of sexual violence against male children and adolescents. An increasing trend was found for Brazil as a whole (annual percentage change = 6.8; 95%CI 3.8;10.0). Spatial distribution showed direct correlation between high rates of violence and low MHDIs (p < 0.001).

**Conclusion:**

We found a rising temporal trend in Brazil and spatial dependence of the rates of reported sexual violence in the municipalities.

## Study contributions

**Main results:**

The trend of reported cases of sexual violence against male children and adolescents was rising in Brazil. The spatial analysis showed a direct correlation between high rates of violence and low human development indexes.

**Implications for services:**

The identification of underreported areas requires the structuring of health surveillance services. Territories with the highest number of reports require prevention and coping strategies to attend to these victims.

**Perspectives:**

Strategies for prevention, coping and improvement of the surveillance process must be adopted. These include: reception, psychosocial support, comprehensive health care and training of health professionals to notify and report the problem.

## RESUMEN

**Objetivo:**

Analizar la tendencia temporal y la distribución espacial de los casos reportados por violencia sexual en niños y adolescentes varones y su relación con el desarrollo municipal en Brasil.

**Métodos:**

Se trata de un estudio ecológico que utiliza datos del Sistema de Información de Enfermedades de Declaración Obligatoria y de Índices de Desarrollo Humano Municipal (IDHM), en el período 2013-2022. Se utilizó la regresión de Prais-Winsten para el análisis temporal y el índice de Moran para el análisis espacial.

**Resultados:**

Hubo 39.967 denuncias de violencia sexual contra niños y adolescentes varones. En Brasil hubo una tendencia creciente (variación porcentual anual = 6,8; IC_95_% 3,8;10,0). La distribución espacial mostró una correlación directa entre altas tasas de violencia y bajos IDHM (p < 0,001).

**Conclusión:**

Se observó una tendencia de crecimiento temporal en Brasil y una dependencia espacial de las tasas de notificación de violencia sexual en los municipios.

## INTRODUCTION

Global prevalence of sexual violence is heterogeneous and does not show a significant decrease, which requires effective coping strategies.¹ One in 13 men reports having been sexually abused between the ages of zero and 17.^2^ Sexual violence against children and adolescents is underestimated for males, due to less understanding or recognition of cases against males.^1^

In Brazil, from 2015 to 2021, 202,948 cases of sexual violence against children and adolescents were recorded on the Notifiable Health Conditions Information System (*Sistema de Informação de Agravos de Notificação* - SINAN). Males had the lowest proportion of cases (13.8%) – following the global trend –, a percentage that could be even higher due to underreporting.^3,4^ This fact is due to the existence of sociocultural barriers, such as *machismo* and discrediting of victims.^4^

The COVID-19 pandemic also contributed to underreporting, with a reduction in reports of this form of violence against children and adolescents in 2020 in the Brazilian state of Rio Grande do Sul.^5^ This trend was also found in North America, Europe, Asia and Africa, even with the exacerbation of sexual abuse.^6^

Tackling sexual violence against male children and adolescents requires understanding the scale of the problem. Trend and spatial analysis studies help services understand the magnitude of a public health problem, in addition to comparisons of exceptional periods, such as the COVID-19 pandemic.^7,8^

Male children and adolescents have been little studied in these analyses. Observing the trend of sexual violence among this population and identifying priority areas is strategic for planning public prevention and coping policies, especially in exceptional periods, such as the pandemic. The objective of this study was to analyze the temporal trend and the spatial distribution of reported cases of sexual violence against male children and adolescents and their relationship with municipal development in Brazil, from 2013 to 2022. 

## METHODS 

### Design and background 

This is an ecological study of time series and spatial analysis. The temporal analysis unit was made up of years and the spatial analysis units were the Brazilian municipalities. 

We used reports of sexual violence against male children and adolescents in Brazil, from 2013 to 2022. This period was chosen because it represents the most recent decade with data on the problem made available by the Information Technology Department of the Brazilian National Health System (*Departamento de Informática do Sistema* Único *de Saúde* - DATASUS).^9^

In 2022, the male population corresponded to 49% of the Brazilian population, and people up to 19 years old corresponded to 14% of this population.^10^

### Participants

We analyzed reported cases of sexual violence against male children and adolescents aged zero to 19 held on the SINAN. We used World Health Organization (WHO) criteria to define the age range of the study, whereby individuals aged zero to 9 are considered children, and individuals aged 10 to 19 are considered adolescents.^11^

### Variables

The study variables were: male sex, age group (in years: < 1, 1-4, 5-9, 10-14, 15-19), region, municipality, year reported, relationship with the aggressor (family member, affective, acquaintance, stranger, person with an institutional relationship, police officer/law enforcement officer, other relationships), place of occurrence (residence, collective housing, school, sports facility, bar or similar, public thoroughfare and commerce/services, industry/construction, other). 

### Data source and measurement 

The number of cases of sexual violence against male children and adolescents was obtained from the SINAN database, and population estimates were obtained from the Brazilian Institute of Geography and Statistics (*Instituto Brasileiro de Geografia e Estatística* - IBGE), available, respectively, on the DATASUS Tabnet platform (https://datasus.saude.gov.br/) and on the 2022 Census website (https://censo2022.ibge.gov.br), obtained on 11/16/2023.^9^

In the case of the “aggressor relationship” variable, the data were aggregated as follows: father, mother, stepfather, stepmother, son, daughter, brother and sister were aggregated as a “Family member” relationship. Spouse, ex-spouse, boyfriend, ex-boyfriend, girlfriend and ex-girlfriend were aggregated as an “Affective” relationship. Friends/acquaintances, carers and bosses were aggregated as an “Acquaintance” relationship. 

The mapping data source (territorial grids) used to generate the maps was the IBGE 2021 version.^12^ We used the 2010 Census^13^ for the Human Development Index (HDI) estimates, given the unavailability of estimates in the 2022 Census.

To calculate the rates of sexual violence against male children and adolescents, the total number of cases per year was divided by the resident population in the respective age groups of each region and of Brazil as a whole in the same year, and multiplied by 100,000.

### Statistical methods

We calculated annual percentage change (APC) in rates of sexual violence against male children and adolescents, based on the Prais-Winsten generalized linear analysis model, using Stata version 14.0.^8^

Initially, we performed log base 10 transformation of the sexual violence rates, with the aim of minimizing the heterogeneity of the variance of the residuals of the regression model, in addition to favoring the calculation of the time series annual growth ratio.^8^ The Prais-Winsten model was used to estimate 95% confidence intervals (95%CI).

The spatial analyses were performed using GeoDa and QGis. Spatial empirical Bayesian rates were calculated for data analysis and representation in maps.^14^ The maps were divided into three-year periods, using natural breaks as a matrix representation.

The spatial autocorrelation analysis was performed using the univariate and bivariate versions of the global and local Moran index.^15^ The univariate models used Bayesian rates of sexual violence against male children and adolescents in isolation, while in the bivariate model, possible spatial correlations between the rates and Municipal Human Development Indexes (MHDIs) were tested. 

To estimate the statistical significance of the indices, the pseudo-significance test was adopted using 99999 permutations.^16^ Cartographic representation was achieved by using the map of local spatial association indicators, which categorizes municipalities based on the local Moran index into direct autocorrelation clusters (high-high and low-low) and inverse correlation clusters (high-low and low-high). 

The Universal Transverse Mercator system was used for the map projections, employing the Geocentric Reference System for the Americas 2000 model. The Moran index calculation were performed using GeoDa version 1.20; QGis versão 3.64 was used for final map layout and creation.

### Ethical aspects

Secondary and aggregated publicly accessible data were used, making it impossible to identify individual records, which exempted the study from being assessed by a research ethics committee. 

## RESULTS

A total of 39,967 cases of sexual violence against male children and adolescents were reported on the SINAN in Brazil during the period studied. Among the reported cases, 16,497 (41.3%) were aged between 5 and 9 years; 14,021 (35.1%) were committed by acquaintances and 11,385 (28.5%) by family members; 24,948 (62.4%) occurred at home; and 17,658 (44.2%) occurred in the Southeast region of Brazil (Table 1). The “repeated violence” variable could not be analyzed due to high incompleteness (31.1%).

**Table 1:** Characterization of reports of sexual violence against male children and adolescents, Brazil, 2013-2022 (n = 39,967)

Variables	N	%
**Age group (years)**		
< 1	724	1.8
1-4	10,277	25.7
5-9	16,497	41.3
10-14	9,224	23.1
15-19	3,245	8.1
**Relationship**		
Family member	11,385	28.7
Affective	346	0.9
Acquaintance	14,021	35.3
Stranger	3,842	9.7
Person with institutional relationship	786	2.0
Police officer/law enforcement officer	71	0.2
Other relationships	9,239	23.3
Unknown	277	0.7
**Place of occurrence**		
Residence	24,948	62.4
Collective housing	619	1.5
School	2,299	5.8
Sports facility	274	0.7
Bar or similar	211	0.5
Public thoroughfare	2,188	5.5
Commerce/services	334	0.8
Industry/construction	105	0.3
Other	4,290	10.7
Unknown	4,699	11.8
**Regions**		
North	4,519	11.3
Northeast	4,549	11.4
Southeast	17,658	44.2
South	9,353	23.4
Midwest	3,888	9.7

**Figure 1:**
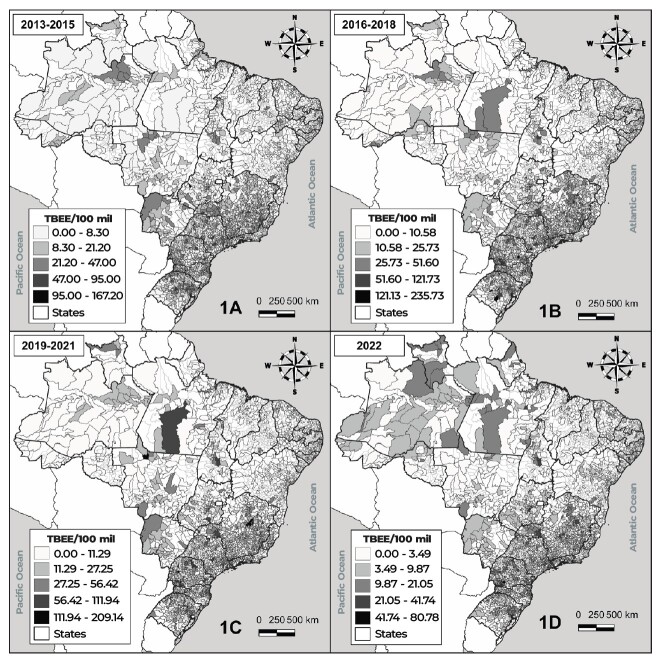
Distribution of reported sexual violence against male child and adolescents in the Brazilian municipalities, 2013-2022

**Figure 2:**
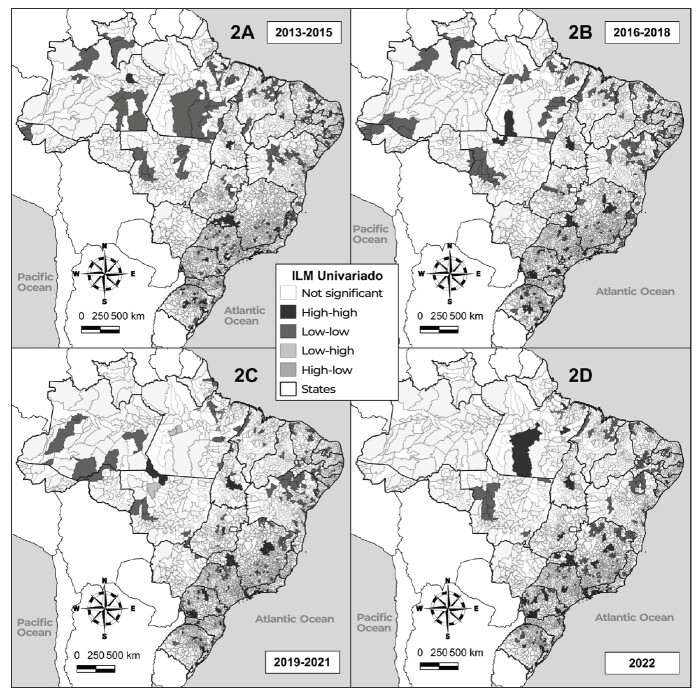
Spatial dependence of reported sexual violence against male child and adolescents in the Brazilian municipalities, 2013-2022

A statistically significant rising trend (APC: 6.8; 95%CI 3.8;10.0; p-value < 0.001) was found for Brazil as a whole (Table 2). APC was higher in the Southeast region (APC: 9.5; 95%CI 5.3;13.9; p-value: 0.001) and Northeast region (APC: 6.8; 95%CI 2.8;11.0) regions ; p-value: 0.004), and among those between 15 and 19 years old (APC: 11.6; 95%CI 7.7;15.6; p-value: < 0.001) and < 1 year old (APC: 10.4; 95%CI 3.8;17.6; p-value: 0.006). Figure 1 and Figure 2

**Table 2:** Annual percentage change (APC) in sexual violence among male children and adolescents (per 100,000 inhabitants), by age group and regions, Brazil, 2013-2022

Age group (years)	APC^a^ (95%CI)^b^	p-value	Trend
**North**			
< 1	13.6 (6.6;21.1)	0.002	rising
1-4	0.8 (-2.3;3.9)	0.586	stationary
5-9	0.9 (-1.3;3.1)	0.370	stationary
10-14	2.6 (0.3;5.0)	0.034	rising
15-19	5.1 (-0.4;11.0)	0.066	stationary
< 1-19	1.8 (-0.2;3.8)	0.070	stationary
**Northeast**			
< 1	12.0 (-3.1;29.4)	0.109	stationary
1-4	7.3 (2.5;12.3)	0.007	rising
5-9	5.4 (1.9;8.9)	0.007	rising
10-14	5.9 (-0.2;12.4)	0.058	stationary
15-19	12.0 (8.3;15.8)	< 0.001	rising
< 1-19	6.8 (2.8;11.0)	0.004	rising
**Southeast**			
< 1	10.7 (1.4;21.0)	0.029	rising
1-4	9.3 (5.9;12.9)	< 0.001	rising
5-9	7.0 (2.2;12.0)	0.010	rising
10-14	10.8 (7.0;14.6)	< 0.001	rising
15-19	13.7 (7.2;20.6)	0.001	rising
< 1-19	9.5 (5.3;13.9)	0.001	rising
**South**			
< 1	14.0 (6.9;21.6)	0.002	rising
1-4	5.1 (1.3;9.0)	0.014	rising
5-9	2.6 (-10.9;6.5)	0.150	stationary
10-14	7.2 (2.3;12.4)	0.009	rising
15-19	10.5 (5.6;15.7)	0.001	rising
< 1-19	5.6 (1.8;9.5)	0.009	rising
**Midwest**			
< 1	7.5 (-4.5;1.9)	0.195	stationary
1-4	4.3 (-1.8;10.7)	0.147	stationary
5-9	1.9 (-2.1;6.1)	0.315	stationary
10-14	7.7 (3.7;11.8)	0.002	rising
15-19	13.5 (5.2;22.4)	0.005	rising
< 1-19	4.9 (0.8;9.1)	0.024	rising
**Brazil**			
< 1	10.4 (3.8;17.6)	0.006	rising
1-4	6.8 (3.8;10.0)	< 0.001	rising
5-9	4.5 (1.3;7.9)	0.012	rising
10-14	8.0 (4.8;11.2)	< 0.001	rising
15-19	11.6 (7.7;15.6)	< 0.001	rising
< 1-19	6.8 (3.8;10.0)	< 0.001	rising

a) APC: Annual percentage change; b) 95%CI: 95% confidence interval.

In the 2013-2015 three-year period, higher rates were concentrated in municipalities in the South and Southeast of Brazil. In the 2016-2018 and 2019-2021 three-year periods, violence rates remained concentrated in the municipalities of the South and Southeast and also in the Northern region state of Pará. In 2022, a differentiated spatial distribution was found in the three-year periods, especially in the municipalities of the Northern region states of Amazonas, Roraima and Amapá, where the rate of sexual violence varied up to 21.05 per 100,000 inhabitants.

There was direct correlation in all years analyzed: 2013-2015 (I: 0.371 p < 0.001), 2016-2018 (I: 0.330 p < 0.001), 2019-2021 (I: 0.391 p < 0.001) and 2022 (I: 0.405 p < 0.001). Clusters of municipalities with high rates of sexual violence were found in the South and Southeast regions and in the Northern region states of Tocantins, from 2013 to 2021 and in 2022, and Pará, from 2013 to 2018 and in 2022.

**Figure 3:**
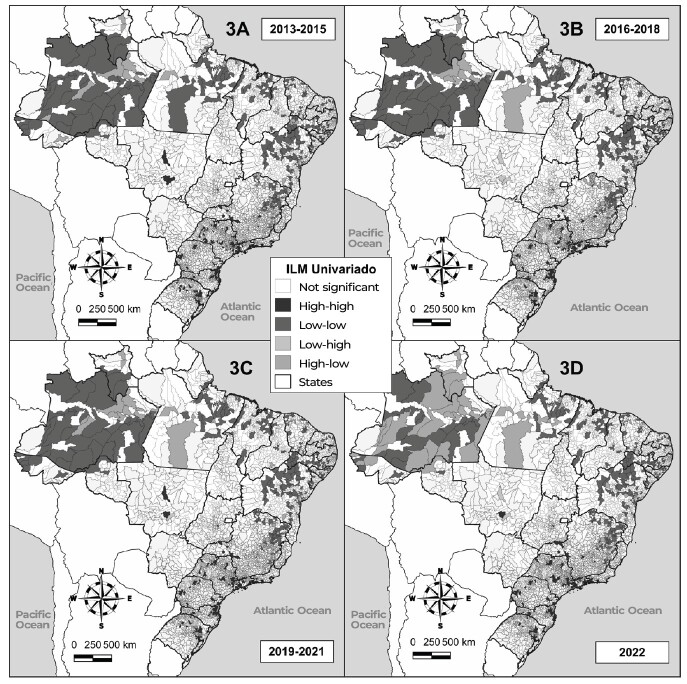
Correlation of reported sexual violence against male children and adolescents according to the human development index in the Brazilian municipalities, 2013-2022

In Figure 3 it can be seen that the bivariate model showed direct global dependence for the four periods analyzed: 2013-2015 (I: 0.196 p < 0.001), 2016-2018 (I: 0.169 p < 0.001), 2019-2021 (I: 0.176 p < 0.001) and 2022 (I: 0.162 p < 0.001). Between 2013 and 2021, there was a predominance of clusters with low rates of sexual violence with low MHDIs. In the South and Southeast regions, especially São Paulo, prevalence of low-high and high-high clusters can be seen. In 2022, the Northern region states of Amazonas, Roraima and Pará concentrated the largest number of municipalities with high rates of sexual violence and low MHDIs. 

## DISCUSSION

Reported cases of violence against male children and adolescents were predominant in children aged 5 to 9 years. The most frequent relationships with the cases were acquaintances or family members. The home was the most frequent place of occurrence. With the exception of the Northern region, the trend was rising throughout Brazil, mainly in the Southeast region. Cases were concentrated in the South and Southeast, while the lowest rates were recorded in the Northeast. Spatial distribution showed correlation with the MHDIs, with emphasis on the states of Amazonas, Roraima and Pará.

A study carried out in the municipality of Rio das Ostras, Rio de Janeiro, revealed that, among cases reported between 2009 and 2018, half were related to children aged 5 to 9 years of both sexes.^17^ In Envigado, Colombia, among 807 cases of sexual violence between 2011 and 2020, 63% occurred in individuals aged 1 to 17.^18^ Due to physical and psychological immaturity, the younger the child, the greater the risk of suffering violence and damage to their health, as they do not have the physical or emotional capacity to defend themselves from such violence.^18^

Acquaintance and family member aggressors were predominant in this study. In Belém, between 2014 and 2016, of 4,870 cases of sexual violence against male children and adolescents, the aggressors of 59% of them were people known to the victims.^19^

The study conducted in Envigado, cited above, revealed that the main aggressors have a family member relationship with the victim, this being a circumstance that makes it difficult to seek help.^18^ Violent experiences, child abuse, mental disorders, drug use, family member dysfunction and limited supervision of children contribute to aggressors having easier access to the victim, especially when they are family members.^18^

The home was the main place of occurrence, which can be associated with the aggressor’s relationship to the victim, since, in most cases, the aggressor is a member of the family or an acquaintance, exercising power over the victim and persisting with sexual abuse. Home should be a welcoming environment for children and adolescents, however, ease of access contributes to the occurrence of sexual violence, mostly in the victim’s home.^17,20^

The Southeast had a greater number of cases, trend and spatial concentration, a result similar to that found by a study characterizing child sexual abuse, from 2011 to 2017, in Brazil.^21^ Greater surveillance present in the country’s large urban centers, through encouragement of reporting and increase in protection services, as in the Southeast region, may influence the number of reports.^21^ In turn, the absence or reduction of case reporting, health services and population adherence to services may explain why the North and Northeast regions had lower rates of sexual violence against male children and adolescents in our study.^21,22^

Between 2013 and 2022, sexual violence against male children and adolescents increased in Brazil and was related to low development. A concentration of municipalities with high rates of violence and low levels of human development was found in Amazonas, Roraima and Pará. Sexual exploitation was closely linked to precarious socioeconomic conditions in empirical research carried out in Manaus, capital of the state of Amazonas, between 2018 and 2019.^23^ Children and adolescents in situations of socioeconomic vulnerability may be sexually exploited as a means of sustenance.^23^ Although violence is not restricted to individuals in vulnerable situations, there is a relationship between sexual abuse and socioeconomic and cultural aspects.^24^

The study has limitations, such as underreporting and incomplete data, which suggest failures in health services to adequately report on the SINAN. Actions such as training health professionals to manage sexual violence and raising community awareness about reporting can help monitor cases. This study contributes to a better understanding of the phenomenon of sexual violence and the development of interventions in line with needs.

## References

[1] Borumandnia N, Khadembashi N, Tabatabaei M, Majd HA (2020). The prevalence rate of sexual violence worldwide: a trend analysis. BMC Public Health.

[2] World Health Organization (WHO) (2022). Child maltreatment. Fact sheets. 2022.

[3] Ministério da Saúde (BR) (2024). Secretaria de Vigilância em Saúde. Boletim Epidemiológico.

[4] Ferreira DC, Bortoli MC, Pexe-Machado P, Saggese SR, Veras MC (2023). Violência sexual contra homens no Brasil: subnotificação, prevalência e fatores associados. Revista de Saúde Pública.

[5] Levandowski ML, Stahnke DN, Munhoz TN, Hohendorff JV, Salvador-Silva R (2021). Impacto do distanciamento social nas notificações de violência contra crianças e adolescentes no Rio Grande do Sul, Brasil. Cadernos de Saúde Pública.

[6] Kourti A, Stavridou A, Panagouli E, Psaltopoulou T, Spiliopoulou C, Tsolia M (2023). Domestic Violence During the COVID-19 Pandemic: A Systematic Review. Trauma, violence, & abuse.

[7] Eryando T (2022). Spatial Analysis for Enhancing the Use of Health Data Availability from Different Sources to Help the Decision-Making Process. Kesmas: Jurnal Kesehatan Masyarakat Nasional (National Public Health Journal).

[8] Antunes JL, Cardoso MR (2015). Uso da análise de séries temporais em estudos epidemiológicos. Epidemiologia e Serviços de Saúde.

[9] Ministério da Saúde (BR) (2022). Datasus: Tabnet - informações de saúde, epidemiológicas e morbidade.

[10] Instituto Brasileiro de Geografia e Estatística (IBGE) (2023). Ministério do Planejamento, Orçamento e Gestão.

[11] World Health Organization (WHO) (2010). Health topics: adolescent health.

[12] Instituto Brasileiro de Geografia e Estatística (IBGE) (2023). Ministério do Planejamento, Orçamento e Gestão.

[13] Instituto de Pesquisa Econômica Aplicada – IPEA (2010). Ipeadata: Índice de Desenvolvimento Humano Municipal (Atlas DH - Censo) [Internet].

[14] Carvalho AXY, Silva GDM, Almeida GR, Albuquerque PHM (2012). Taxas bayesianas para o mapeamento de homicídios nos municípios brasileiros. Cadernos de Saúde Pública.

[15] Luzardo AJR, Castañeda RM, Rubim IB (2017). Análise espacial exploratória com o emprego do índice de Moran. GEOgraphia.

[16] Druck S, Carvalho MS, Câmara G, Monteiro AMV (2004). Análise Espacial de Dados Geográficos.

[17] Barcellos TMT, Góes FGB, Silva ACSS, Souza AN, Camilo LA, Goulart MCL (2021). Violência contra crianças: descrição dos casos em município da baixada litorânea do Rio de Janeiro. Escola Anna Nery.

[18] Noreña-Herrera C, Rodríguez SA (2022). Violencia sexual en un municipio de Colombia: características de las víctimas y de sus victimarios, 2011-2020. Biomédica.

[19] Ferraz MMP, Veloso MMX, Cabral IR (2021). Violência sexual contra crianças e adolescentes: análise das notificações a partir do debate sobre gênero.

[20] Silva SBJ, Conceição HN, Câmara JT (2020). Perfil das notificações de violência contra crianças e adolescentes. Revista de Enfermagem UFPE Online.

[21] Cândido EL, Girão MMF, Assunção RCG, Feitosa PWG, Oliveira IC, Oliveira IC (2020). Características do abuso sexual infantil no brasil. Revista Feminismos.

[22] Moreira KFA, Bicalho BO, Moreira TL (2020). Violência sexual contra mulheres em idade fértil na região norte do Brasil. Revista Eletrônica Acervo Saúde.

[23] Fernandez CB, Silva SEP (2020). Acompanhamento especializado de adolescentes em situação de violência sexual na cidade de Manaus. Revista de Políticas Públicas.

[24] Miranda AC, Barreto MLM, Lírio VS, Clemente F (2020). Violência sexual contra crianças e adolescentes no Brasil: uma revisão sistemática da literatura. Ciências Sociais Unisinos.

