# Supplementary material for: Temporal and spatial analysis of notifications of sexual violence against male children and adolescents in Brazil, 2013 to 2022: an ecological study
Source: Epidemiol Serv Saude. 2024 Oct 14;33:e20231439. doi: 10.1590/S2237-96222024v33e20231439.en (PMC11473067; doi:10.1590/S2237-96222024v33e20231439.en)
Supplement: Translation

# Translation: Análise temporal e espacial das notificações de violência sexual contra crianças e adolescentes do sexo masculino no Brasil, 2013 a 2022: estudo ecológico

**DOI:** 10.1590/S2237-96222024v33e20231439.pt

**Authors:** Beatriz Caroline Leão Lima, Cássio Eduardo Soares Miranda, Fernando Ferraz do Nascimento, Jesusmar Ximenes Andrade, Malvina Thais Pacheco Rodrigues, José Wicto Pereira Borges

**Affiliations:** 1 Universidade Federal do Piauí, Programa de Pós-Graduação em Saúde e Comunidade, Teresina, PI, Brasil

**Keywords:** Violência Sexual, Abuso Sexual de Crianças e Adolescentes, Estudos Ecológicos, Estudos de Séries Temporais, Análise Espacial

## Abstract

**Objetivo:**

Analisar a tendência temporal e a distribuição espacial dos casos notificados de violência sexual contra crianças e adolescentes do sexo masculino, e sua relação com o desenvolvimento municipal no Brasil.

**Métodos:**

Trata-se de estudo ecológico empregando dados do Sistema de Informação de Agravos de Notificação e os Índices de Desenvolvimento Humano Municipal (IDHMs), de 2013-2022. Utilizou-se a regressão de Prais-Winsten para a análise temporal e o índice de Moran para a análise espacial.

**Resultados:**

Houve 39.967 notificações de violência sexual contra crianças e adolescentes do sexo masculino. Verificou-se tendência crescente no Brasil (variação percentual anual = 6,8; IC_95_% 3,8;10,0). A distribuição espacial apresentou correlação direta entre altas taxas de violência e baixos IDHMs (p < 0,001).

**Conclusão:**

Observou-se tendência temporal de crescimento no Brasil e dependência espacial das taxas de notificação de violência sexual nos municípios.

## Contribuições do estudo

**Principais resultados:**

A tendência dos casos notificados de violência sexual contra crianças e adolescentes do sexo masculino foi crescente no Brasil. A análise espacial apresentou correlação direta entre altas taxas de violência e baixos índices de desenvolvimento humano.

**Implicações para os serviços:**

A identificação de áreas subnotificadas requer estruturação de serviços de vigilância em saúde. Territórios com maior número de notificações demandam estratégias de prevenção e enfrentamento para o acolhimento dessas vítimas.

**Perspectivas:**

Estratégias de prevenção, enfrentamento e melhoria do processo de vigilância devem ser adotadas. Incluem-se entre elas: acolhimento, suporte psicossocial, assistência integral à saúde e treinamento de profissionais para notificação e denúncia do agravo.

## INTRODUÇÃO

A prevalência mundial da violência sexual é heterogênea e sem diminuição significativa, o que requer estratégias de enfrentamento eficazes.^1^ Um a cada 13 homens relata ter sido abusado sexualmente entre zero e 17 anos de idade.^2^ A violência sexual contra crianças e adolescentes é subestimada para o sexo masculino, devido à menor compreensão ou reconhecimento dos casos contra os homens.^1^

No Brasil, de 2015 a 2021, foram registrados 202.948 casos de violência sexual em crianças e adolescentes no Sistema de Informação de Agravos de Notificação (Sinan). O sexo masculino teve a menor proporção de casos (13,8%) – seguindo a tendência mundial –, percentual que pode ser ainda maior, devido à subnotificação.^3,4^ Tal fato se deve à existência de barreiras socioculturais, como machismo e descredibilização das vítimas.^4^

A pandemia de covid-19 também contribuiu para a subnotificação, com redução de notificações dessa violência contra crianças e adolescentes em 2020 no Rio Grande do Sul.^5^ Tal comportamento foi observado também na América do Norte, Europa, Ásia e África, mesmo com exacerbação dos abusos sexuais.^6^

O enfrentamento da violência sexual contra crianças e adolescentes do sexo masculino requer a compreensão da dimensão do problema. Estudos de tendência e análise espacial auxiliam os serviços no conhecimento da magnitude de um problema de saúde pública, além da comparação em períodos excepcionais, como na pandemia de covid-19.^7,8^

Nessas análises, crianças e adolescentes do sexo masculino têm sido pouco estudados. Observar a tendência da violência sexual nesse público e identificar áreas prioritárias é estratégico para o planejamento de políticas públicas de prevenção e enfrentamento, principalmente em períodos excepcionais, como na pandemia. O objetivo deste estudo foi analisar a tendência temporal e a distribuição espacial dos casos notificados de violência sexual contra crianças e adolescentes do sexo masculino, e sua relação com o desenvolvimento municipal no Brasil, de 2013 a 2022. 

## MÉTODOS 

### Delineamento e contexto

Trata-se de estudo ecológico de série temporal e análise espacial. A unidade de análise temporal foi composta pelos anos e a unidade de análise espacial foi composta pelos municípios. 

Utilizaram-se as notificações de violência sexual contra crianças e adolescentes do sexo masculino no Brasil, de 2013 a 2022. Esse período foi escolhido por representar a década mais recente com dados sobre o agravo disponibilizados pelo Departamento de Informática do Sistema Único de Saúde (DATASUS).^9^

Em 2022, a população masculina correspondia a 49% da população brasileira, e pessoas até 19 anos correspondiam a 14% dessa população.^10^

### Participantes

Foram analisados os casos notificados de violência sexual contra crianças e adolescentes do sexo masculino de zero a 19 anos notificados no Sinan. Para a definição da faixa etária do estudo, utilizou-se o critério da Organização Mundial da Saúde (OMS), em que indivíduos de zero a 9 anos são considerados crianças, e indivíduos de 10 a 19 anos, adolescentes.^11^

### Variáveis

As variáveis do estudo foram: sexo masculino, faixa etária (em anos: < 1, 1-4, 5-9, 10-14, 15-19), região, município, ano da notificação, vínculo do agressor (familiar, afetivo, conhecido, desconhecido, pessoa com relação institucional, policial/agente da lei, outros vínculos), local de ocorrência (residência, habitação coletiva, escola, local de prática esportiva, bar ou similar, via pública e comércio/serviços, indústria/construção, outros). 

### Fonte de dados e mensuração 

O número de casos de violência sexual contra crianças e adolescentes do sexo masculino foi obtido a partir da base de dados do Sinan, e as estimativas populacionais, a partir do Instituto Brasileiro de Geografia e Estatística (IBGE), disponíveis respectivamente na plataforma Tabnet do DATASUS (https://datasus.saude.gov.br/) e no sítio eletrônico do Censo de 2022 (https://censo2022.ibge.gov.br), obtidos em 16/11/2023.^9^

Para a variável “vínculo do agressor”, foi realizada a agregação dos dados: pai, mãe, padrasto, madrasta, filho(a) e irmão(a) foram agregados como vínculo “Familiar”. Cônjuge, ex-cônjuge, namorado(a) e ex-namorado(a) foram agregados como vínculo “Afetivo”. Amigos/conhecidos, cuidador e patrão/chefe foram agregados como vínculo “Conhecido”. 

A fonte de dados da base cartográfica (malhas territoriais) utilizada para a geração dos mapas é proveniente do IBGE, na versão de 2021.^12^ A estimativa do IDH utilizada é a do Censo de 2010,^13^ tendo em vista a indisponibilidade de estimativas no Censo de 2022.

Para o cálculo das taxas de violência sexual contra crianças e adolescentes do sexo masculino, foi dividido o número total de casos por ano pela população residente nas respectivas faixas etárias de cada região e do país no mesmo ano, e multiplicado por 100 mil.

### Métodos estatísticos

Foi calculada a variação percentual anual (VPA) das taxas de violência sexual contra crianças e adolescentes do sexo masculino, baseada no modelo de análise linear generalizada de Prais-Winsten, usando-se o programa Stata versão 14.0.^8^

Inicialmente, foi realizada a transformação logarítmica na base 10 das taxas de violência sexual, com o objetivo de minimizar a heterogeneidade da variância dos resíduos do modelo de regressão, além de favorecer o cálculo da razão de incremento anual da série temporal.^8^ O modelo de Prais-Winsten foi empregado na estimação dos intervalos de confiança de 95% (IC_95_%).

As análises espaciais foram realizadas com uso do GeoDa e do QGis. Foram calculadas as taxas bayesianas empíricas espaciais para análise de dados e representação em mapas.^14^ Os mapas foram divididos em triênios, utilizando-se como representação matricial as quebras naturais.

A análise de autocorrelação espacial foi realizada pelo índice global e local de Moran em suas versões univariada e bivariada.^15^ Os modelos univariados utilizaram isoladamente as taxas bayesianas de violência sexual contra crianças e adolescentes do sexo masculino, enquanto no bivariado foram testadas possíveis correlações espaciais entre as taxas e os Índices de Desenvolvimento Humano Municipal (IDHMs). 

Para estimar a significância estatística dos índices, foi adotado o teste de pseudossignificância por meio de 99.999 permutações.^16^ A demonstração cartográfica foi realizada por meio do mapa de indicadores locais de associação espacial, que categoriza os municípios a partir do índice local de Moran em agrupamentos de autocorrelação direta (alto-alto e baixo-baixo) e de correlação inversa (alto-baixo e baixo-alto). 

A projeção dos mapas utilizou o sistema Universal Transversa de Mercator, empregando o modelo Sistema de Referência Geocêntrico para as Américas 2000. Os cálculos dos índices de Moran foram realizados no GeoDa versão 1.20; a diagramação e a criação dos mapas finais foram realizadas no QGis versão 3.64.

### Aspectos éticos

Foram utilizados dados secundários e agregados de acesso público, impossibilitando a identificação individual dos registros, o que dispensou o estudo de apreciação por comitê de ética em pesquisa. 

## RESULTADOS

Foram notificados no Sinan 39.967 casos de violência sexual contra crianças e adolescentes do sexo masculino no Brasil, no período estudado. Entre os casos notificados, 16.497 (41,3%) eram da faixa etária de 5 a 9 anos; 14.021 (35,1%) eram de vínculo conhecido e 11.385 (28,5%) de vínculo familiar; 24.948 (62,4%) tiveram a residência como local de ocorrência; e 17.658 (44,2%) ocorreram na região Sudeste (Tabela 1). A variável “violência de repetição” não pôde ser analisada, devido à elevada incompletude (31,1%). Figura 1 e Figura 2

**Figura 1:** Distribuição das notificações de violência sexual contra crianças e adolescentes do sexo masculino nos municípios brasileiros, 2013-2022

**Figura 2:** Dependência espacial das notificações de violência sexual contra crianças e adolescentes do sexo masculino nos municípios brasileiros, 2013-2022

**Tabela 1:** Caracterização das notificações por violência sexual contra crianças e adolescentes do sexo masculino, Brasil, 2013-2022 (n = 39.967)

Variáveis	N	%
**Faixa etária (anos)**		
< 1	724	1,8
1-4	10.277	25,7
5-9	16.497	41,3
10-14	9.224	23,1
15-19	3.245	8,1
**Vínculo**		
Familiar	11.385	28,7
Afetivo	346	0,9
Conhecido	14.021	35,3
Desconhecido	3.842	9,7
Pessoa com relação institucional	786	2,0
Policial/agente da lei	71	0,2
Outros vínculos	9.239	23,3
Ignorado	277	0,7
**Local de ocorrência**		
Residência	24.948	62,4
Habitação coletiva	619	1,5
Escola	2.299	5,8
Local de prática esportiva	274	0,7
Bar ou similar	211	0,5
Via pública	2.188	5,5
Comércio/serviços	334	0,8
Indústria/construção	105	0,3
Outros	4.290	10,7
Ignorado	4.699	11,8
**Regiões**		
Norte	4.519	11,3
Nordeste	4.549	11,4
Sudeste	17.658	44,2
Sul	9.353	23,4
Centro-Oeste	3.888	9,7

Observou-se tendência crescente estatisticamente significativa (VPA: 6,8; IC_95_% 3,8;10,0; p-valor < 0,001) no Brasil (Tabela 2). A VPA foi maior nas regiões Sudeste (VPA: 9,5; IC_95_% 5,3;13,9; p-valor: 0,001) e Nordeste (VPA: 6,8; IC_95_% 2,8;11,0; p-valor: 0,004), e entre 15 e 19 anos (VPA: 11,6; IC_95_% 7,7;15,6; p-valor: < 0,001) e < 1 ano (VPA: 10,4; IC_95_% 3,8;17,6; p-valor: 0,006).

**Tabela 2:** Variação percentual anual (VPA) da violência sexual em crianças e adolescentes do sexo masculino (por 100 mil habitantes), pela faixa etária e regiões, Brasil, 2013-2022

Faixa etária (anos)	VPA^a^ (IC_95_ **%**)^b^	p-valor	Tendência
**Norte**			
< 1	13,6 (6,6;21,1)	0,002	crescente
1-4	0,8 (-2,3;3,9)	0,586	estacionária
5 a 9	0,9 (-1,3;3,1)	0,370	estacionária
10 a 14	2,6 (0,3;5,0)	0,034	crescente
15 a 19	5,1 (-0,4;11,0)	0,066	estacionária
< 1 a 19	1,8 (-0,2;3,8)	0,070	estacionária
**Nordeste**			
< 1	12,0 (-3,1;29,4)	0,109	estacionária
1 a 4	7,3 (2,5;12,3)	0,007	crescente
5 a 9	5,4 (1,9;8,9)	0,007	crescente
10 a 14	5,9 (-0,2;12,4)	0,058	estacionária
15 a 19	12,0 (8,3;15,8)	< 0,001	crescente
< 1 a 19	6,8 (2,8;11,0)	0,004	crescente
**Sudeste**			
< 1	10,7 (1,4;21,0)	0,029	crescente
1 a 4	9,3 (5,9;12,9)	< 0,001	crescente
5 a 9	7,0 (2,2;12,0)	0,010	crescente
10 a 14	10,8 (7,0;14,6)	< 0,001	crescente
15 a 19	13,7 (7,2;20,6)	0,001	crescente
< 1 a 19	9,5 (5,3;13,9)	0,001	crescente
**Sul**			
< 1	14,0 (6,9;21,6)	0,002	crescente
1 a 4	5,1 (1,3;9,0)	0,014	crescente
5 a 9	2,6 (-10,9;6,5)	0,150	estacionária
10 a 14	7,2 (2,3;12,4)	0,009	crescente
15 a 19	10,5 (5,6;15,7)	0,001	crescente
< 1 a 19	5,6 (1,8;9,5)	0,009	crescente
**Centro-Oeste**			
< 1	7,5 (-4,5;1,9)	0,195	estacionária
1 a 4	4,3 (-1,8;10,7)	0,147	estacionária
5 a 9	1,9 (-2,1;6,1)	0,315	estacionária
10 a 14	7,7 (3,7;11,8)	0,002	crescente
15 a 19	13,5 (5,2;22,4)	0,005	crescente
< 1 a 19	4,9 (0,8;9,1)	0,024	crescente
**Brasil**			
< 1	10,4 (3,8;17,6)	0,006	crescente
1 a 4	6,8 (3,8;10,0)	< 0,001	crescente
5 a 9	4,5 (1,3;7,9)	0,012	crescente
10 a 14	8,0 (4,8;11,2)	< 0,001	crescente
15 a 19	11,6 (7,7;15,6)	< 0,001	crescente
< 1 a 19	6,8 (3,8;10,0)	< 0,001	crescente

a) VPA: Variação percentual anual; b) IC_95_%: Intervalo de confiança de 95%.

No triênio 2013-2015, maiores taxas se concentraram em municípios do Sul e Sudeste do país. Nos triênios 2016-2018 e 2019-2021, as taxas de violência permaneceram concentradas nos municípios do Sul e Sudeste, e no estado do Pará. Em 2022, observou-se uma distribuição espacial diferenciada nos triênios, especialmente nos municípios do Amazonas, de Roraima e do Amapá, em que a taxa de violência sexual apresentou variação de até 21,05 por 100 mil habitantes.

Houve correlação direta em todos os anos analisados: 2013-2015 (I: 0,371 p < 0,001), 2016-2018 (I: 0,330 p < 0,001), 2019-2021 (I: 0,391 p < 0,001) e 2022 (I: 0,405 p < 0,001). Agrupamentos de municípios com altas taxas de violência sexual foram verificados nas regiões Sul e Sudeste e nos estados do Tocantins, de 2013 a 2021 e em 2022, e do Pará, de 2013 a 2018 e em 2022.

A Figura 3 mostra que o modelo bivariado apresentou dependência global direta para os quatro períodos analisados: 2013-2015 (I: 0,196 p < 0,001), 2016-2018 (I: 0,169 p < 0,001), 2019-2021 (I: 0,176 p < 0,001) e 2022 (I: 0,162 p < 0,001). Entre os anos de 2013 e 2021, observou-se predominância de agrupamentos de baixas taxas de violência sexual com baixos IDHMs. Nas regiões Sul e Sudeste, especialmente em São Paulo, é possível notar prevalência de agrupamentos baixo-alto e alto-alto. Em 2022, os estados do Amazonas, de Roraima e do Pará concentraram a maior quantidade de municípios cujas taxas de violência sexual eram altas e os IDHMs eram baixos. 

**Figura 3:** Correlação entre as notificações de violência sexual contra crianças e adolescentes do sexo masculino e o índice de desenvolvimento humano nos municípios brasileiros, 2013-2022

## DISCUSSÃO

Os casos notificados de violência contra crianças e adolescentes do sexo masculino foram predominantes em crianças de 5 a 9 anos. Os vínculos mais frequentes dos casos eram conhecidos ou familiares. A residência foi o local mais constante das ocorrências. Excetuando-se a região Norte, a tendência foi crescente em todo o Brasil, principalmente na região Sudeste. Os casos se concentraram no Sul e Sudeste, enquanto as menores taxas se registraram no Nordeste. A distribuição espacial apontou correlação com os IDHMs, com destaque para os estados do Amazonas, de Roraima e do Pará.

Estudo realizado no município de Rio das Ostras, Rio de Janeiro, revelou que, entre as notificações de 2009 a 2018, metade eram referentes a crianças de 5 a 9 anos de idade de ambos os sexos.^17^ Em Envigado, Colômbia, entre 807 casos de violência sexual entre 2011 e 2020, 63% ocorreram em indivíduos de 1 a 17 anos.^18^ Devido à imaturidade física e psicológica, quanto mais nova a criança, maior o risco de sofrer violência e danos à saúde, pois ela não possi capacidade física e emocional para se defender da violência.^18^

Agressores conhecidos e familiares foram predominantes neste estudo. Em Belém, entre 2014 e 2016, de 4.870 casos de violência sexual contra crianças e adolescentes do sexo masculino, 59% tinham como agressores pessoas conhecidas das vítimas.^19^

O estudo de Envigado, citado acima, revelou que os principais agressores têm vínculo familiar com a vítima, circunstância que dificulta a busca de ajuda.^18^ Experiências violentas, abuso infantil, transtornos psicopatológicos, uso de drogas, disfunção familiar e vigilância limitada das crianças contribuem para o acesso facilitado dos agressores à vítima, principalmente quando são familiares.^18^

A residência foi o principal local de ocorrências, o que pode ser associado ao vínculo do agressor, visto que, na maioria dos casos, o agressor é membro da família ou conhecido, exercendo poder sobre a vítima e persistindo nos abusos sexuais. O lar deveria representar um ambiente acolhedor para crianças e adolescentes, entretanto, a facilidade de acesso contribui para a ocorrência da violência sexual majoritariamente na residência da vítima.^17,20^

O Sudeste mostrou maior número de casos, tendência e concentração espacial, resultado semelhante ao de um estudo de caracterização do abuso sexual infantil, de 2011 a 2017, no Brasil.^21^ A maior vigilância presente nos grandes centros do país, por meio de incentivo à notificação e aumento de serviços de proteção, como ocorre na região Sudeste, pode influenciar no número de notificações.^21^ Por seu turno, a ausência ou a redução de notificação de casos, de serviços de saúde e da adesão da população aos serviços pode explicar por que as regiões Norte e Nordeste apresentaram taxas de violência sexual contra crianças e adolescentes do sexo masculino inferiores, no presente estudo.^21,22^

Entre 2013 e 2022, a violência sexual contra crianças e adolescentes do sexo masculino aumentou no Brasil e se relacionou a baixo desenvolvimento. Observou-se concentração de municípios com altas taxas de violência e baixos índices de desenvolvimento humano no Amazonas, em Roraima e no Pará. A exploração sexual apresentou-se intimamente ligada às condições socioeconômicas precárias em uma pesquisa empírica realizada em Manaus, entre 2018 e 2019.^23^ Crianças e adolescentes em situação de vulnerabilidade socioeconômica podem ser exploradas sexualmente como forma de sustento.^23^ Apesar de a violência não se restringir a indivíduos em situação de vulnerabilidade, há relação do abuso sexual com aspectos socioeconômicos e culturais.^24^

O estudo apresenta limitações, como a subnotificação e a incompletude de dados, que sugerem falhas nos serviços de saúde para a notificação adequada no Sinan. Ações como o treinamento de profissionais para o manejo da violência sexual e a sensibilização da comunidade para a denúncia podem auxiliar na vigilância dos casos. O estudo contribui para a melhor compreensão do fenômeno e o desenvolvimento de intervenções consentâneas às necessidades.
